# Hematological alterations associated with long COVID-19

**DOI:** 10.3389/fphys.2023.1203472

**Published:** 2023-07-24

**Authors:** Guilherme C. Lechuga, Carlos M. Morel, Salvatore Giovanni De-Simone

**Affiliations:** ^1^ Center for Technological Development in Health (CDTS)/ National Institute of Science and Technology for Innovation in Neglected Population Diseases (INCT-IDPN), Oswaldo Cruz Foundation (FIOCRUZ), Rio de Janeiro, Brazil; ^2^ Laboratory of Epidemiology and Molecular Systematics (LESM), Oswaldo Cruz Institute, Oswaldo Cruz Foundation (FIOCRUZ), Rio de Janeiro, Brazil; ^3^ Laboratory of Cellular Ultrastructure, Oswaldo Cruz Institute, Oswaldo Cruz Foundation (FIOCRUZ), Rio de Janeiro, Brazil; ^4^ Post-Graduation Program in Science and Biotechnology, Department of Molecular and Cellular Biology, Biology Institute, Federal Fluminense University, Niterói, Brazil

**Keywords:** long COVID-19, hematology, post-acute sequelae, SARS-CoV-2, red blood cells, COVID-19

## Abstract

Long COVID-19 is a condition characterized by persistent symptoms lasting beyond the acute phase of COVID-19. Long COVID-19 produces diverse symptomatology and can impact organs and systems, including the hematological system. Several studies have reported, in COVID-19 patients, hematological abnormalities. Most of these alterations are associated with a higher risk of severe disease and poor outcomes. This literature review identified studies reporting hematological parameters in individuals with Long COVID-19. Findings suggest that Long COVID-19 is associated with a range of sustained hematological alterations, including alterations in red blood cells, anemia, lymphopenia, and elevated levels of inflammatory markers such as ferritin, D-dimer, and IL-6. These alterations may contribute to a better understanding of the pathophysiology of Long COVID-19 and its associated symptoms. However, further research is needed to elucidate the underlying mechanisms and potential treatments for these hematological changes in individuals with Long COVID-19.

## 1 Introduction

The COVID-19 pandemic, caused by the SARS-CoV-2 virus, spread rapidly and significantly impacted societies and economies worldwide. Although efforts are ongoing to control the virus’s spread and reduce its effects, the successful vaccination strategy significantly decreased morbidity and mortality (Hadj Hassine, 2022). However, the emergence of SARS-CoV-2 variants due to viral mutations produced deep concern in the population. Due to increased transmissibility, some variants became dominant. Furthermore, the spread of Omicron variants, which are more transmissible, demonstrated that these variants also increased resistance to neutralization and vaccination (Hoffmann et al., 2022).

The disease primarily affects the respiratory system. However, COVID-19 can also impact other organs and systems, including the hematological system. Several studies have reported hematological abnormalities in COVID-19 patients. Some alterations include an increase in white blood cell count, a decrease in red blood cell count and hemoglobin levels, an increase in ferritin levels, increase in levels of D-dimer and other markers of coagulation (Słomka et al., 2020; Ye et al., 2020; Al-Saadi and Abdulnabi, 2022; Gajendra, 2022). These abnormalities are associated with a higher risk of severe disease and worse outcomes (Hariyanto et al., 2021).

The symptoms of COVID-19 can range from mild to severe and can include fever, cough, shortness of breath, fatigue, body aches, loss of taste or smell, and sore throat. It is important to highlight that not all COVID-19 patients experience hematological abnormalities, and the severity of these abnormalities can vary widely among patients (Gajendra, 2022).

Some symptoms can persist for months after the initial infection with COVID-19. In this case, the disease is known as Long COVID, or post-acute sequelae of SARS-CoV-2 infection (PASC). These symptoms can occur even in individuals who have mild or asymptomatic infections. Although Long COVID’s effects are still under investigation, the symptoms vary widely. They can include fatigue, shortness of breath, chest pain, joint pain, headaches, brain fog, difficulty concentrating, loss of taste or smell, and mood disorders. Neurological damages are mainly associated with Long COVID (Proal and VanElzakker, 2021). However, people may also experience organ damage, such as heart, lung, or kidney problems (Davis et al., 2023).

Evidence suggests that age, sex, and race can influence the development and severity of Long COVID. Long COVID affects both males and females, but some studies indicate that females have a higher risk (Bai et al., 2022; Perlis et al., 2022; Subramanian et al., 2022). In addition, older individuals are generally more likely to experience persistent symptoms and prolonged recovery (Bai et al., 2022; Perlis et al., 2022). Certain racial and ethnic groups have differences in cognitive symptomology associated with Long COVID (Jacobs et al., 2023). A recent meta-analysis showed that female sex, age, high body mass index, and smoking were associated with an increased risk of developing Long COVID (Tsampasian et al., 2023).

The pathophysiological mechanisms of Long COVID are still under debate and include the effect of immune response to the virus, inflammation, and autoimmune response (Davis et al., 2023). Evidence suggests that the hematological system is altered in Long COVID, with reports of lower hemoglobin levels and increased D-dimer levels that could lead to blood clotting frequently associated with Long COVID (Lehmann et al., 2021). Also, some patients with Long COVID-19 displayed anemia, thrombocytopenia, and lymphopenia (Pasini et al., 2021; Sonnweber et al., 2022). The subsequent sections of this review provide a comprehensive analysis of the hematological alterations in COVID-19 and Long COVID and possible pathological mechanisms.

## 2 Long COVID-19

Long COVID, or PASC, is a multisystem disorder with multiple persistent or new symptoms (Davis et al., 2023), affecting 10%–30% of infected individuals. However, the exact pathophysiology remains poorly understood. According to the latest definition, Long COVID is considered if symptoms and abnormalities are present beyond 12 weeks of acute COVID-19. Symptoms between weeks 4–12 are defined as ‘sub-acute’ or ‘ongoing symptomatic COVID-19'(Haunhorst et al., 2022).

This morbidity has been associated with debilitating systemic disorders such as cardiovascular disease, cerebrovascular disease, thrombotic events and coagulopathy, type 2 diabetes, and myalgic encephalomyelitis/chronic fatigue syndrome (ME/CFS) (Davis et al., 2021; 2023; Peghin et al., 2021; Xie and Al-Aly, 2022; Xie et al., 2022). Symptoms can persist for years, and for some, symptoms can be expected to be lifelong. Recently, it was suggested that Long COVID could have a more deleterious effect on society and economics. Neurocognitive impairment was linked to loss of productivity and unemployment (Perlis et al., 2023). Neurocognitive disorders are frequently found in PASC, occurring in approximately 70% of individuals. Dysfunction varies from brain fog, depression, anxiety, headaches, insomnia, dizziness, anosmia, and dysgeusia (Davis et al., 2021; Graham et al., 2021; Guo et al., 2022).

**FIGURE 1:**
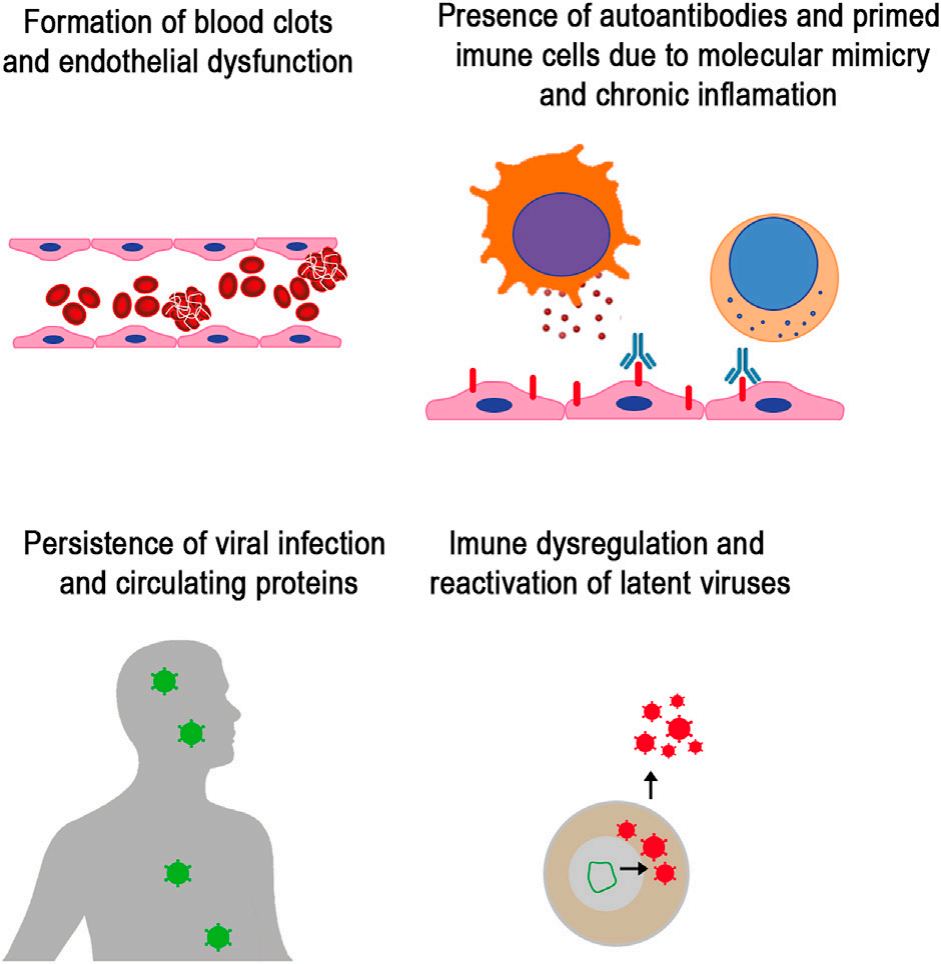
Mechanism involved in Long COVID. Studies have identified the main factor in persistent symptoms that characterize Long COVID. Mechanisms include vascular dysfunction and formation of micro clots that lead to thrombosis, immune dysregulation with increased pro-inflammatory response and autoreactive immunity driven by molecular mimicry and bystander activation of lymphocytes, the persistence of viral replication and SARS-CoV-2 proteins circulation, and reactivation of human latent herpes viruses.

Multiple factors may overlap to cause Long COVID. Several hypotheses for its pathogenesis have been suggested (Figure 1). One proposed mechanism is the persistent presence of SARS-CoV-2 in tissues (Sumi and Harada, 2022). In addition, the study demonstrated the presence of Spike protein after 1 year of infection (Swank et al., 2023).

COVID-19 is an immune-mediated disease, and dysregulation of the immune system is also present in Long COVID. Individuals with Long COVID had persistent immune dysregulation, including increased levels of inflammatory cytokines and decreased T cells and B cells (Shuwa et al., 2021)and dysregulation of innate and adaptative immune cells population (Ryan et al., 2022). It was observed that individuals with Long COVID had higher levels of autoantibodies compared to healthy controls (Rojas et al., 2022) and sex-matched patients with other respiratory infections (Son et al., 2022). Molecular mimicry and bystander lymphocyte activation could explain the autoimmune-driven Long COVID hypothesis. Evidence that cytokines could activate CD8^+^ cell populations without involving TCR has been proposed to explain post-acute COVID-19 complications (Gregorova et al., 2020; Churilov et al., 2022). In addition, the reactivation of latent pathogens, including herpesviruses and others, may contribute to Long COVID (Peluso et al., 2023). Immune-mediated vascular dysfunction is another mechanism proposed in Long COVID that leads to persistent microvascular blood clotting, thrombosis, and thromboembolism (Pretorius et al., 2021). A persistent capillary rarefaction was observed in Long COVID, even after 18 months (Osiaevi et al., 2023). Also, a study demonstrated changes in the size and morphology of red blood cells in Long COVID that potentially can affect oxygen diffusion (Kubánková et al., 2021; Grau et al., 2022). Blood biomarkers can potentially predict Long COVID status and aid the treatment and medical intervention. Changes in hematological parameters and blood biomarkers persist in Long COVID (Brundyn et al., 2022).

## 3 Hematological alterations in sub-acute and long COVID-19

Different blood alterations occur in COVID-19 and are a predictor of possible biomarkers for outcome and treatment. However, for most infected individuals, hematological parameters return to normal within days after disease onset. However, this parameter remains elevated for a fraction of infected individuals for months and even years. These hematological markers, in some cases, are associated with PASC. Here we list the most relevant alteration in hematological parameters associated with Long COVID (Table 1). The following discussions included studies evaluating hematological alterations in sub-acute (symptoms between 4–12 weeks) and Long COVID-19 (symptoms present beyond 12 weeks).

**TABLE 1:** Summary table of most frequent hematological biomarkers of Long COVID.

Hematological parameter	Status in long COVID
Cells and cellular parameters	
RBC	↓(Kubánková et al., 2021; Grau et al., 2022; Lin et al., 2022)
MCV	↑(Lin et al., 2022)
MCHC	↑(Alfadda et al., 2022)
Lymphocytes	↓ (Mandal et al., 2021; Moreno-Pérez et al., 2021; Alfadda et al., 2022); ↔(Darcis et al., 2021)
Blood components and proteins	
Hemoglobin	↓(Kubánková et al., 2021; Pasini et al., 2021; Grau et al., 2022; Pereira-Roche et al., 2022); ↔ (Darcis et al., 2021; Alfadda et al., 2022)
Platelet count	↔ (Mandal et al., 2021; Pasini et al., 2021; Alfadda et al., 2022)
D-dimer	↑ (Mandal et al., 2021; Moreno-Pérez et al., 2021; Pasini et al., 2021; Fan et al., 2022; Kalaivani and Dinakar, 2022)
Ferritin	↑ (Moreno-Pérez et al., 2021; Pasini et al., 2021); ↔(Darcis et al., 2021; Mandal et al., 2021; Alfadda et al., 2022; Pérez-González et al., 2022)
C-reactive protein (C.R.P.)	↑ (Mandal et al., 2021; Pasini et al., 2021; Pérez-González et al., 2022); ↔ (Darcis et al., 2021; Kalaivani and Dinakar, 2022)
Lactate dehydrogenase	↔(Moreno-Pérez et al., 2021; Pasini et al., 2021; Alfadda et al., 2022)
Cytokines	
IL-6	↑ (Fan et al., 2022; Schultheiß et al., 2022); ↓(Williams et al., 2022); ↔ (Kalaivani and Dinakar, 2022; Queiroz et al., 2022)
TNF-α	↑(Peluso et al., 2021; Schultheiß et al., 2022); ↔ (Queiroz et al., 2022; Williams et al., 2022)
IL-1β	↑(Schultheiß et al., 2022); ↔ (Williams et al., 2022)
IL-2	↓ (Williams et al., 2022); ↑ (Queiroz et al., 2022)
IL17	↓ (Williams et al., 2022); ↑(Queiroz et al., 2022)
IFNγ	↓(Williams et al., 2022); ↔ (Queiroz et al., 2022)
IFN-β	↑(Phetsouphanh et al., 2022)
IFN-λ1	↑(Phetsouphanh et al., 2022)
IL-8	↓(Williams et al., 2022); ↔ (Schultheiß et al., 2022); ↑(Phetsouphanh et al., 2022)
IL-10	↔ (Williams et al., 2022); ↓(Queiroz et al., 2022)
IL-4	↓(Queiroz et al., 2022; Williams et al., 2022); ↔(Schultheiß et al., 2022)

Note: ↔ denotes no significant change to a reference value or control group, while ↑ denotes an increase and ↓ denotes a decrease in the parameter.

### 3.1 Cells

A decrease in lymphocyte count is a common feature in Long COVID (Mandal et al., 2021; Moreno-Pérez et al., 2021; Alfadda et al., 2022). In COVID-19, lymphopenia is a predictor of severity (Illg et al., 2021), and the reduction of T lymphocytes is unusual in viral infections. It is believed that in severe COVID-19, deficient interferon production driven by SARS-CoV-2 can impair T cells, as interferons are important to promote survival and effector functions of T cells (Sa Ribero et al., 2020; Proal and VanElzakker, 2021). Lymphopenia can be caused by direct viral infection since lymphocytes express Angiotensin-converting enzyme 2 (ACE2) (Xu et al., 2020), cytokine storm with a significant increase of IL-6 induce lymphopenia (Tang et al., 2020; Montazersaheb et al., 2022) and lymphocytic infiltration to organs (Proal and VanElzakker, 2021). T-cell exhaustion is also believed to contribute to SARS-CoV-2 persistence (Ramakrishnan et al., 2021).

Alteration in red blood cells (RBC) can occur in COVID-19 (Russo et al., 2022), and despite most studies showing that in the Long COVID normal range is recovered for most individuals (Darcis et al., 2021; Mandal et al., 2021; Moreno-Pérez et al., 2021; Alfadda et al., 2022), evidence suggests a phenotypic change that could be linked to Long COVID (Grau et al., 2022). Pasini et al. (2021) showed a high degree of erythrocyte sedimentation after 2 months of follow-up. Determination of RBC parameters after an average of 60.7 days shows that hemoglobin concentration, mean corpuscular volume (MCV), and mean corpuscular hemoglobin (MCH), were highly altered in COVID-19 and RBC deformability was significantly reduced in post-COVID-19 patients (Grau et al., 2022). Indeed, RBC morphology changes were observed in patients discharged after 4 and 8 months. Alterations include decreased size and deformability of erythrocytes of hospitalized and recovered COVID-19 patients (Kubánková et al., 2021).

These altered physical properties reflect the changes in plasma membranes and cytoskeleton networks. The interaction between SARS-CoV-2 and RBC occurs via the Band-3 and the spike protein, and in the bone marrow, the virus interacts with nascent erythroblasts through CD147 and CD26 (Wang et al., 2020; Kronstein-Wiedemann et al., 2022). Changes in RBC include modifications in shape, size, and deformability, which can alter microvascular perfusion, endothelial cell integrity, blood flow behavior, and hemostasis (Kubánková et al., 2021; Nader et al., 2022; Russo et al., 2022). Reduced deformability of RBCs increases their likelihood of adhering to the vessel wall, resulting in elevated vascular resistance and risk of thrombosis (Weisel and Litvinov, 2019). In addition, vascular endothelial damage can be caused by long-term viral infection, chronic hypoxia, and inflammation (Wang et al., 2022). Also, alteration in RBC structure and metabolism linked to high shear rates, inflammation, and oxidative stress activate scramblase externalizing phosphatidylserine (P.S.) on the outer membrane, P.S. provides a scaffold for coagulation cascade (Whelihan and Mann, 2013; Weisel and Litvinov, 2019), enhances RBC adherence and activation of the endothelium, and increase RBC-microvesicle secretion that enhances the hypercoagulability state (Kim et al., 2018; Leal et al., 2018).

The decrease in deformability can lead to impaired rheology and hemolysis, RBCs from COVID-19 patients may be particularly susceptible to the attack of reactive oxygen species (R.O.S.), leading to cell lysis and reduced oxygen-carrying capacity. The RBC rigidity increases hemolysis and releases free hemoglobin molecules that scavenge nitric oxide leading to platelet activation (Rother et al., 2005; Diederich et al., 2018). During acute SARS-CoV-2 infection, damaged endothelial cells are critical in promoting diffuse microthrombi formation and disrupting various endothelial barriers throughout the body. These microthrombi contribute to multiple organ dysfunction (Wu et al., 2023). These alterations could impair proper circulation, promote hypoxia, and favor coagulopathies, common in Long COVID (Gillespie and Doctor, 2021).

Understanding these complex interactions and their impact on RBCs is crucial for comprehending the hematological changes associated with Long COVID-19, particularly concerning vascular effects and disease severity.

Individuals who followed for 6 months, with at least one Long COVID-19-related symptom, had a significantly higher mean corpuscular hemoglobin concentration (MCHC) than those who recovered with no signs (Alfadda et al., 2022). Higher MCHC can have multiple causes, including autoimmune hemolytic anemia, and rare events have been reported in COVID-19 (Lazarian et al., 2020; Al-Mashdali et al., 2021; Fattizzo et al., 2021).

Changes in blood parameters can be evidenced after 2 years. Most hematologic indicators regarding WBC and platelet counts in COVID-19 convalescents were comparable to those of the healthy control group. However, RBC counts, hemoglobin, red blood cell distribution width-coefficient of variation, and mean corpuscular hemoglobin showed statistical differences. Although indicators related to RBC showed recovery to the normal range, RBC counts were abnormal in 26.4% (20/76) after 1 year and 8.5% (5/59) after 2 years of disease onset. Interestingly, the 2-year follow-up showed the proportion of mean corpuscular volume (MCV) above the normal range increased significantly from 2.6% (2/76) to 37.3% (22/59) (Lin et al., 2022).

### 3.2 Blood components and proteins

Hemoglobin is responsible for gas exchange in RBCs. COVID-19 hemoglobin levels were reported to be low, compromising oxygen transport of RBCs in COVID-19 patients leading to hypoxia and is related to disease severity (Anai et al., 2021). Several hypotheses were elaborated to explain this viral effect. First, it was evidenced that SARS-CoV-2 can interact with RBC via receptors CD147 and Band 3 (Cosic et al., 2020; Wang et al., 2020), and several structural, including Spike and nucleoprotein and non-structural proteins, can bind hemoglobin and heme (Lechuga et al., 2021), possibly altering protein function. Second, the virus scavenges some molecules like bilirubin and biliverdin, a heme and hemoglobin degradation product, to evade antibodies (Rosa et al., 2021). Third, SARS-CoV-2 was found to infect erythroid precursor cells derived from peripheral CD34^+^ blood stem cells and disrupt hemoglobin biosynthesis (Kronstein-Wiedemann et al., 2022). Another possibility is that the virus triggers an immune response that causes inflammation, the release of cytokines, and oxidative stress, which can lead to the breakdown of RBCs and the release of hemoglobin into the bloodstream. This can decrease hemoglobin levels, induce hypoxia and anemia (Russo et al., 2022). The chronic hypoxia in Long COVID is mostly related to lung function impairment (Caruso et al., 2021; Cueto-Robledo et al., 2022), but it also provides enhanced release of inflammatory cytokines (Østergaard, 2021). Under the hypoxic stimulus, changes in the erythroid precursor maturation of reticulocytes and young RBC occur. They seem to have low catalase, and increased ROS formation contributes to the preferential destruction of young RBC upon return to normoxia (Risso et al., 2007; Song et al., 2015). These events can promote anemia, inflammation, and iron deficiency in Long COVID (Sonnweber et al., 2022).

In Long COVID, hemoglobin levels tend to return to normal values, but some reports showed that for some patients, this parameter is still altered (Pasini et al., 2021; Pereira-Roche et al., 2022; Lai et al., 2023). Generally, Long COVID symptoms have multiple causes, but low hemoglobin levels and anemia can contribute to fatigue, weakness, and shortness of breath.

Alterations of iron homeostasis can persist in Long COVID-19, markedly hyperferritiemia. Ferritin is a protein composed of two subunits, H and L. Its main function is to store iron, regulating cellular oxygen metabolism (Plays et al., 2021). Ferritin expression and upregulation can be triggered by inflammation and oxidative stress. Serum ferritin levels are increased in COVID-19 due to inflammation and the release of cytokines, particularly IL-6, that stimulate hepcidin synthesis, a master regulator of iron uptake and distribution (Ganz, 2011). Ferritin has immune modulatory functions, mediating inflammation and exerting immunosuppressive effects on T and B cells (Kernan and Carcillo, 2017). Elevated serum levels of ferritin have been found to correlate with the severity of COVID-19 (Kaushal et al., 2022). Iron overload can lead to oxidative stress, lipid peroxidation, and, ultimately, cell death by ferroptosis (Winterbourn, 1995; Girelli et al., 2021).

Additionally, ferritin-increased levels have been linked to interactions between RBCs and platelets in COVID-19 patients, suggesting a role in thrombosis in COVID-19 (Venter et al., 2020) and possibly in Long COVID. After 60 days of follow-up, hyperferritinemia remained elevated in 38% of individuals and was more frequent in patients with severe disease (Pasini et al., 2021). Additionally, persisting ferritin elevation correlated with severe lung disease and iron dysmetabolism contributed to impaired stress resilience at long-term COVID-19 follow-up (Sonnweber et al., 2022).

An analysis of hematological changes in long-COVID19 revealed that in some patients, the increased D-dimer levels are sustained for several months. D-dimer is produced by fibrin degradation. Elevated levels of D-dimer correlate with COVID-19 severity (Yu et al., 2020). A post-COVID follow-up study showed that 30% of patients had elevated D-dimer approximately 54 days after hospital discharge (Mandal et al., 2021). Another study showed that all 75 patients with previously confirmed COVID-19 had increased D-dimer and ferritin 2 months after hospital discharge (Pasini et al., 2021). After a median of 3 months following COVID-19 patients, 15% still had a persistent D-dimer elevation. It was more frequently associated with patients that had severe COVID-19 (Lehmann et al., 2021). After 4 months, increased D-dimer levels (>500 ng/ml) were observed in 25.3% of convalescent patients, but in most (>90%) of this patient’s other coagulation markers (prothrombin time, activated partial thromboplastin time, fibrinogen, platelet count) had returned to normal values (Townsend et al., 2021). The D-dimer levels start to decrease but continue high for up to 6 months in patients after discharge from the hospital. The persistence of D-dimer elevated levels is associated with Long COVID symptoms (Kalaivani and Dinakar, 2022). Indeed, thromboembolic complications are a common feature of Long COVID. In this viral infection, the coagulation pathway is activated due to the immune response and cytokine storm leading to the hypercoagulable and pro-inflammatory state (Lazzaroni et al., 2021). RBC could also play a role in the cytokine storm since RBC store and release several cytokines, including pro-inflammatory TNF-α and IL-1β(Karsten et al., 2018).

Additionally, intravascular hemolysis could lead to an inflammatory state and excessive cytokine release, due to oxidative imbalance induced by hemoglobin degradation and the release of heme and iron (Bozza and Jeney, 2020). Iron metabolism is directly influenced by cytokines that trigger the production of hepcidin, which binds to ferroportin, restricting the availability of iron and preventing its export to cells (Ganz, 2011). However, in COVID-19, there is an excess of iron within cells and tissues, accompanied by reduced serum iron levels. Iron dysmetabolism could restrict hemoglobin and RBC synthesis, leading to anemia and sustained hypoxia in Long COVID (Cavezzi et al., 2020; Girelli et al., 2021; Russo et al., 2022).

Several works also showed higher levels of C-reactive protein (CRP), an acute phase protein, in Long COVID (Mandal et al., 2021; Pasini et al., 2021). CRP is produced in the liver after stimulating inflammatory cytokines like IL-6 (Sproston and Ashworth, 2018). This biomarker is sustained increased and is possibly linked to cytokine sustained elevation in Long COVID (Lai et al., 2023). The enzyme lactate dehydrogenase (LDH) is a mark of tissue damage. Levels of this enzyme in some post-acute COVID-19 are also increased. After 2 months of infection, LDH levels were elevated in more than 27% of subjects (Pasini et al., 2021). Multiple factors may contribute to long-term COVID cell lysis, including viral persistence and immune dysregulation (Davis et al., 2023).

### 3.3 Cytokines

During COVID-19, virus infection induces an intense production of cytokines, the “cytokine storm.” This response is implicated in triggering immunopathological reactions. Various cytokines (IFN-γ, IL-1β, IL-6, IL-2, and TNF-α) had altered levels in COVID-19, and the cytokine storms correlate with the severity and progression of COVID-19 (Huang et al., 2020; Chang et al., 2021). However, studies evaluating cytokine levels in Long COVID are contradictory. A Long COVID cohort of 12 individuals showed reductions in circulating levels of cytokines, remarkably Interferon Gamma (IFNγ) and Interleukin-8 (IL-8). Authors proposed that immune exhaustion drives long-COVID (Williams et al., 2022). SARS-CoV-2 expresses proteins that allow it to counteract the induction or escape the antiviral activity of interferons. Additionally, an inadequate or delayed IFN-I response contributes to the disease (Sa Ribero et al., 2020). In contrast, an increased expression of interferon I (IFN-β) and III (IFN-λ1) was evidenced 8 months after infection. Patients with Long-Covid have higher levels than age- and gender-matched recovered individuals without Long COVID, unexposed donors, and individuals infected with other coronaviruses (Phetsouphanh et al., 2022). The IFN type I and III production imbalance is consistent with the prolonged activation of plasmacytoid dendritic cells, indicating a chronic inflammatory response.

Another study with 135 individuals with PASC revealed that subjects with Long COVID-19 had higher levels of IL-17 and IL-2, and subjects without PASC had higher levels of IL-10, IL-6, and IL- 4 (Queiroz et al., 2022). However, a cohort that followed Long COVID subjects for 8 months observed a different profile. The study showed a long-lasting cytokine signature consisting of elevated levels of interleukin (IL)-1β, IL-6, and tumor necrosis factor (TNF- α) (Schultheiß et al., 2022). Similarly, a study found that IL-6 and TNF-α elevation was sustained in subjects that experienced symptoms at approximately 120 days following COVID-19 (Peluso et al., 2021).

These works confirm that immune dysregulation and sustained pro-inflammatory cytokine production are linked to Long COVID symptomatology. Current data of a cytokine biomarker are relevant as they can serve as diagnostic or prognostic information and be used to monitor Long COVID.

## 4 Conclusion

This paper highlights the hematological alterations associated with Long COVID-19, which may have important implications for diagnosing, monitoring, and treating this condition. A literature review suggests that Long COVID-19 is a respiratory disease and a systemic disorder affecting multiple organs and systems, including the hematopoietic system. Researchers and clinicians should identify the most frequent and relevant hematological alterations and consider monitoring these parameters in individuals with Long COVID-19. Future research should focus on elucidating the underlying mechanisms of these hematological changes and exploring potential therapeutic interventions to improve outcomes in individuals with Long COVID-19. It is important to note that not all Long COVID-19 patients experience hematological abnormalities. The symptomatology and the severity of these abnormalities can also vary widely among patients. Overall, this paper contributes to a better understanding of the multifaceted nature of Long COVID-19 and highlights the importance of a multidisciplinary approach to managing this condition.
